# DNA Repair Gene *ZmRAD51A* Improves Rice and *Arabidopsis* Resistance to Disease

**DOI:** 10.3390/ijms20040807

**Published:** 2019-02-13

**Authors:** Fang Liu, Yunjian Xu, Lingyan Zhou, Asif Ali, Haiyang Jiang, Suwen Zhu, Xiaoyu Li

**Affiliations:** National Engineering Laboratory of Crop Stress Resistance Breeding, Anhui Agricultural University, Hefei 230036, China; weishanren163@163.com (F.L.); xuyunjian1992@163.com (Y.X.); zhoulingyan518@126.com (L.Z.); be_my_lovep@yahoo.com (A.A.); hyjiang@ahau.edu.cn (H.J.); zhusuwen@126.com (S.Z.)

**Keywords:** disease resistance, ZmRAD51A, maize, rice, *Arabidopsis*

## Abstract

RAD51 (DNA repair gene) family genes play ubiquitous roles in immune response among species from plants to mammals. In this study, we cloned the *ZmRAD51A* gene (a member of *RAD51*) in maize and generated *ZmRAD51A* overexpression (*ZmRAD51A*-OE) in rice, tobacco, and *Arabidopsis*. The expression level of *ZmRAD51A* was remarkably induced by salicylic acid (SA) application in maize, and the transient overexpression of *ZmRAD51A* in tobacco induced a hypersensitive response. The disease resistance was significantly enhanced in *ZmRAD51A*- OE (overexpressing) plants, triggering an increased expression of defense-related genes. High-performance liquid chromatography (HPLC) analysis showed that, compared to control lines, *ZmRAD51A*-OE in rice plants resulted in higher SA levels, and conferred rice plants resistance to *Magnaporthe oryzae*. Moreover, the *ZmRAD51A*-OE *Arabidopsis* plants displayed increased resistance to *Pseudomonas syringae* pv. *tomato* DC3000 when compared to wild types. Together, our results provide the evidence that, for the first time, the maize DNA repair gene *ZmRAD51A* plays an important role in in disease resistance.

## 1. Introduction

In plant immune systems, PAMP (pathogen-associated molecular pattern)-triggered immunity (PTI) through pattern recognition is the first line of defense, which keeps most potential invaders in check [1]. Effector-triggered immunity (ETI) is a second line of defense by recognition of attacker-specific effector molecules [1]. The local activation of a PTI or ETI response triggers systemic acquired resistance (SAR) [2]. SAR is accompanied by the increased levels of SAR signal salicylic acid (SA), which then causes the accumulation, nuclear translocation, and turnover of the transcription cofactor NPR1 (non-expressor of PR1 genes), leading to the activation of pathogeneses-related (PR) genes [3,4,5]. SAR provides long-lasting, broad spectrum resistance to secondary infection [6]. Studies have shown that the DNA repaired protein RAD51D and SNI1 (suppressor of NPR1 inducible 1) coregulated NPR1-independent PR gene expression [7]. DNA damage repair proteins SSN2 (Suppressor of *sni1* 2) and RAD51D replace the transcription repressor SNI1 at PR gene promoters, and their coordinated action ensures plant immune gene expression during plant defense [8].

The RAD51 recombination protein was first discovered in *Saccharomyces cerevisiae* [9]. It is homologous to the *Escherichia coli* RecA protein, which plays a vital role in homologous recombination, a well-known repair process of DNA double strand breaks (DSBs) [9]. During the lifetime of plant, DNA damage always occurs, including DSBs, caused by environmental stresses or intercellular events [10]. DSBs influence genome stability and, if not repaired, they can substantially affect cell metabolism and viability [10]. RAD51 was found to express in both meiosis and mitosis and was involved in DNA repair and recombination [11]. Orthologues of *RAD51* genes have been identified in several plant species, including *Arabidopsis thaliana*, *Oryza sativa*, *Zea mays*, *Triticum aestivum*, and *Lycopersicon esculentum*. In *Arabidopsis*, *AtRAD51* is required during meiotic recombination [12] and its mRNA level increases after gamma irradiation [13]. In addition, *AtRAD51B* and *AtRAD51C* are reported to be involved in meiosis, mitosis and DNA repair process in somatic cells [14,15]. In rice, OsRAD51 (rice DNA repair gene) protein promoted homology dependent renaturation, as well as strand exchange reactions, in rice [16]. Another *RAD51* gene of rice, *OsRAD51D*, is a negative factor for telomere lengthening and plays a critical role in reproductive growth in rice [17]. In maize, *RAD51* shows higher levels of expression in mitotic and meiotic tissues [18], and recent studies have indicated that the RAD51 protein is involved in meiotic chromosome synapses and segregation [19,20]. In wheat, *TaRAD51* expression is restricted to meiotic and highly increases during prophase I of meiosis [21]. A *RAD*-like gene of *Gossypium barbadense GbRL1* is involved in cotton early ovule development and/or fiber initiation [22]. Except for the established roles in meiotic recombination, the presence of some *RAD51* has also show the immune function in pathogen invasion; for instance, the *rad51d* mutant of *Arabidopsis* enhances disease susceptibility [7], abolishes *PR* genes transcriptional inducibility, and leads to disease susceptibility [23]. 

Maize (*Zea mays* L.) is one of the most important crops in the world, but it is susceptible to many diseases that cause reduced crop yield. So far, a number of genes involved in maize disease resistance have been identified. For example, *ZmRxo1* and *ZmRp1-D*, two genes containing nucleotide-binding site (NBS), are involved in resistance to diverse pathogen strains and confer resistance to rice bacterial streak disease [24] and rust [25]. *ZmHtn1* encoding a putative wall-associated receptor-like kinase confers partial northern corn leaf blight resistance for maize by delaying the beginning of lesion formation [26]. *RAD51* genes are another class of genes that function in plant disease response. In maize, there are two closely related *RAD51* genes, *ZmRAD51A* and *ZmRAD51B*. Previous studies have indicated that maize *ZmRAD51* can function in the homology search phase of chromosome pairing and meiotic recombination [18], as well as in the repair of MuDR (autonomous mutator transposon)-induced DSBs [27]. However, little is known about the roles of *ZmRAD51* genes in maize immune response. In this study, we isolate *ZmRAD51A* and functionally characterize the role of *ZmRAD51A* in conferring disease resistance to rice and *Arabidopsis*.

## 2. Results

### 2.1. Cloning and Characterization of ZmRAD51A

**Figure 1:**
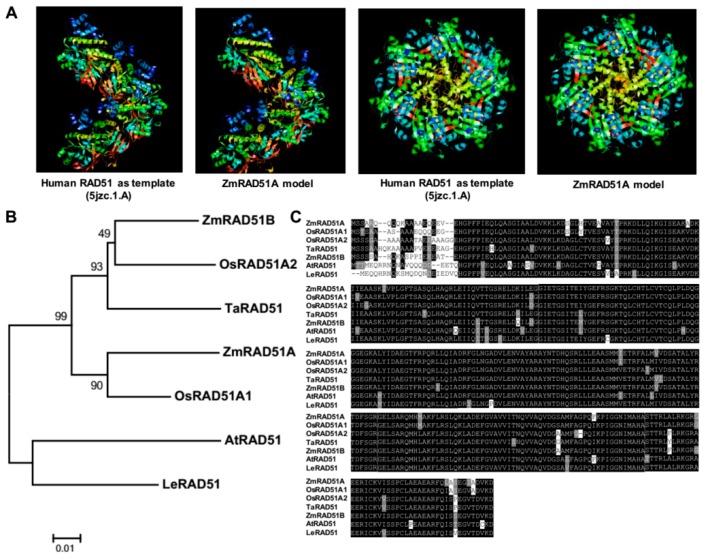
Characterization of ZmRAD51A. (**A**) The 3D structures of the ZmRAD51A model. (**B**) Phylogenetic analysis of ZmRAD51A and other RAD51 proteins from model species. Bootstrap values (1000 replicates) are shown as percentages at the branch nodes. Bar = 0.01. The GenBank accession numbers are: ZmRAD51B (NP_001104919.1) from *Zea mays*, AtRAD51 (OAO95923.1) from *Arabidopsis thaliana*, LeRAD51 (Q40134.1) from *Lycopersicon esculentum*, TaRAD51 (ACM47239.1) from *Triticum aestivum*, OsRAD51A1 (BAB85490.1) and OsRAD51A2 (ABI58231.1) from *Oryza sativa Japonica*. (**C**) Conserved domain comparisons between the amino acid sequence of ZmRAD51A and other RAD51 proteins. Black color represents identical amino acids sequences and gray color represents similar amino acid.

The full-length of *ZmRAD51A* (NP_001104918.2) coding sequence was obtained from the cDNA of maize B73 leaves by using PCR with specific primers. The *ZmRAD51A* transcript has 9 exons, encoding 340 amino acids (Figure S1). The predicted 3D model of ZmRAD51A showed similar structures with that of human RAD51 (Figure 1A). An unrooted phylogenetic tree generated by known RAD51 proteins exhibited a high association between ZmRAD51A and OsRAD51A1, and the two proteins share 92.46% sequence identity (Figure 1B). Amino acid sequence alignment of ZmRAD51A and other RAD51 proteins from *Zea mays*, *Arabidopsis thaliana*, *Lycopersicon esculentum*, *Triticum aestivum* and *Oryza sativa Japonica* suggested conservation of RAD51 across the species (Figure 1C). 

**Figure 2:**
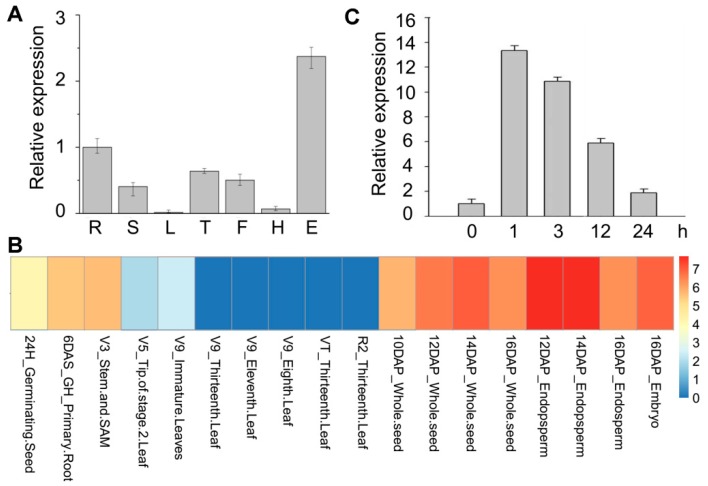
Gene expression patterns of *ZmRAD51A* in maize. (**A**) Spatial expression pattern of *ZmRAD51A* in maize. Data represent means relative expression values + SD. R = root, S = stem, L = leaf, T = tassel, F = flower, H = husk, E = ear. (**B**) Heat map of *ZmRAD51A* gene expression in maize. The expression scale of high, medium, and low are represented as red, yellow, and blue colors at right, respectively. V = vegetative growth stage, R = reproductive growth stage, H = hours, DAS = days after sowing, and DAP = days after pollination, GH = greenhouse, V = vegetative, VT = vegetative tasseling, R = reproductive. (**C**) Gene expression levels of *ZmRAD51A* in leaf samples response to salicylic acid (SA) application. Three-leaf stage maize seedlings received SA and leaves were harvested at 0 h, 1 h, 3 h, 12 h and 24 h after SA treatment. *ZmActin* [28] was used for normalization. The expression levels of 0 h was used as the control and assigned value of 1. Data represent means SD. Three biological replicates were performed.

The spatial expression pattern analysis revealed that *ZmRAD51A* is highly expressed in maize ears, tassels, filaments, roots, and stems, but is relatively lower in leaves (Figure 2A). By employing previously reported transcriptome database, we further investigated the expression profiles of *ZmRAD51A* gene in tissues at different developmental stages. *ZmRAD51A* showed high expression levels in seeds, endosperm, embryos, SAM (shoot apical meristem), and roots (Figure 2B). *ZmRAD51A* was lower expressed in leaves, supported by the qRT-PCR (Quantitative real time polymerase chain reaction) result (Figure 2B). To test whether *ZmRAD51A* expression in maize leaves responds to biotic stress in SA signaling pathways, we applied SA to maize leaves at the three-leaf stage. The transcript level of *ZmRAD51A* increased from 1 to 24 h after SA treatment, where the highest transcript level (~13-fold up-regulation) was observed in the first hour after SA treatment (Figure 2C).

### 2.2. Transient Overexpression of ZmRAD51A in Nicotiana Benthamiana Leaves Induced a Hypersensitive Response 

**Figure 3:**
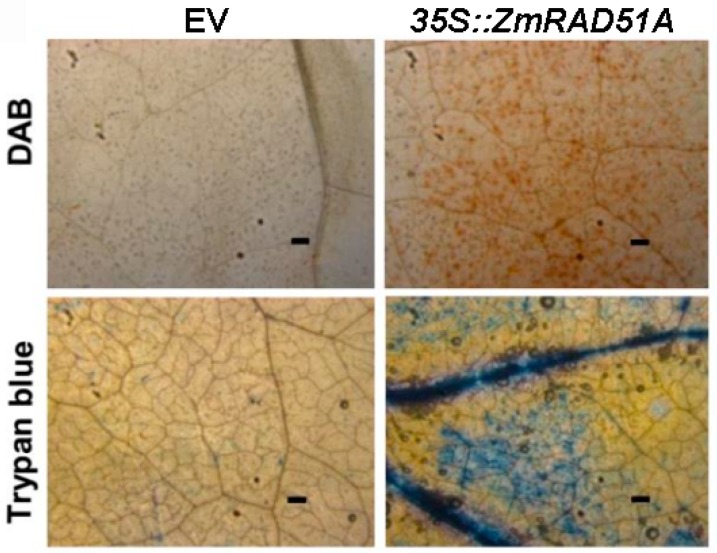
Transient expression of *ZmRAD51A* in *Nicotiana benthamiana* affected immunity induction. DAB (Diaminobenzidine) staining and Trypan blue staining in *N. benthamiana leaves* 48 h after 35S:*ZmRAD51A*-Agrobacterium and pCAMBIA1301 (EV)-Agrobacterium infiltration. Bars = 0.5 mm.

The 35S:*ZmRAD51A* construct, driven by CaMV35S promoter, was generated. The *A. tumefaciens* GV3101 containing 35S:*ZmRAD51A* vector and pCAMBIA1301 (control) infiltrated *N. benthamiana* leaves to verify hypersensitive response cell death. DAB (Diaminobenzidine) staining showed a large amount of H_2_O_2_ was accumulated in transformed *N. benthamiana* leaf overexpressing *ZmRAD51A* after 48 h (Figure 3). Trypan blue staining analysis correlated with the visual damage index [29]. *ZmRAD51A* transient overexpression leaf showed darker trypan blue staining than that of pCAMBIA1301 (Figure 3). These results suggest that transient overexpression of *ZmRAD51A* in tobacco leaves induced a hypersensitive response and H_2_O_2_ accumulation in response to stress.

### 2.3. Transgenic Overexpression of ZmRAD51A Increased SA Synthesis and Conferred Rice Resistance to M. oryzae 

**Figure 4:**
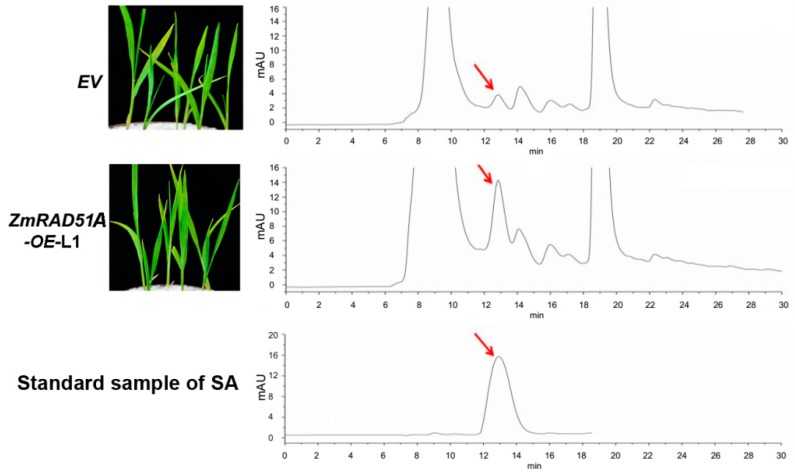
Chromatogram of SA extracted from *ZmRAD51A*-*OE* and pCAMBIA1301 transgenic rice without pathogen inoculation. The SA chromatogram from top to bottom represents pCAMBIA1301 (EV), *ZmRAD51A*-*OE*, and standard SA. The arrow indicates the SA peak; mAU indicates peak height. EV represents pCAMBIA1301.

**Figure 5:**
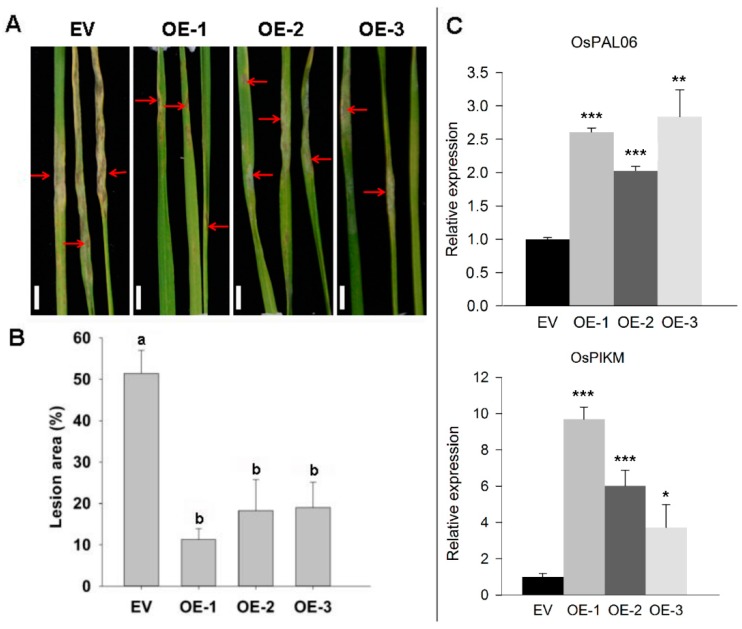
*ZmRAD51A*-*OE* transgenic rice plants enhanced resistance to *M*. *oryzae* at seedling stages. (**A**) Phenotype of *ZmRAD51A*-*OE* and pCAMBIA1301 (EV) transgenic rice at 7 dpi (days post infection) of *M. oryzae*. OE-1, OE-2, OE-3 represent three transgenic lines of *ZmRAD51A*-*OE* transgenic rice plant. Red arrows represent blast lesions. Bars = 50 mm. (**B**) Statistical analysis of lesion area in *M. oryzae* infected leaves. Different letters above the columns indicate significant differences at *p* < 0.05 level among EV, OE-1, OE-2 and OE-3. (**C**) Gene expression patterns of defense-related genes in *ZmRAD51A*-*OE* and pCAMBIA1301 (EV) inoculated with *M. oryzae*. *Student’s* test was performed between pCAMBIA1301 and *ZmRAD51A*-*OE* transgenic lines (* *p* < 0.05, ** *p* < 0.01 and *** *p* < 0.001). *OsActin* [31] was used for normalization. The expression levels of inoculated EV was used as the control and assigned value of 1. Data represent means SD. Three biological replicates were performed.

To test whether *ZmRAD51A* overexpression confers disease resistance in rice, 70 pieces of rice calli were induced and 19 transgenic lines expressing *ZmRAD51A* (*ZmRAD51A*-*OE*) were generated. Three transgenic lines renamed OE-1, 2, and 3, were randomly selected for further analysis. Transgenic expression analysis showed the three transgenic lines had different expression levels (Figure S2). The growth of *ZmRAD51A-OE* transgenic rice at the seedling stage was similar to the empty vector (*EV*) transgenic lines (Figure 4). Demonstrated by high-performance liquid chromatography (HPLC), we found that SA accumulation drastically increased in *ZmRAD51A*-OE plants (peak area 400.596 g^–1^) compared with control plants (peak area 74.749 g^–1^) without any pathogen inoculation (Figure 4). *ZmRAD51A*-*OE* transgenic plants at 4–5-leaf stage were challenged with *M. oryzae*, which can cause rice blast. Interestingly, overexpression of *ZmRAD51A* reduced macroscopic blast symptoms, with fewer and smaller blast lesions than the control at 7 dpi of *M. oryzae* (Figure 5A). *ZmRAD51A*-*OE* plants showed the lesion area ranging from 11.3 (OE-1) to 19.0% (OE-3) at 7 dpi, while that of the control plants was 51.4% (Figure 5B). In addition, the expression levels of rice blast resistant genes, *OsPAL06*, and *OsPIKM* [30], were respectively increased 2.3-fold and 5.1-fold in *ZmRAD51A*-*OE* transgenic lines at 7 dpi of *M. oryzae* (Figure 5C). These results together suggest that the overexpression of *ZmRAD51A* provides an enhanced resistance to rice blast in *ZmRAD51A*-*OE* lines.

To investigate whether *ZmRAD51A* gene affect rice yield, we measured rice yield-related traits, including the seed size and 1000-grain weight, among *EV* control and *ZmRAD51A*-*OE* lines. The seeds of *ZmRAD51A*-*OE* rice showed no significant size (in terms of length and width) and 1000-grain weight when compared to *EV* control rice (Figure S3).

### 2.4. ZmRAD51A Overexpression in Arabidopsis Enhanced Resistance to Pst DC3000 Triggering by Increased SA-Related Genes Expression

**Figure 6:**
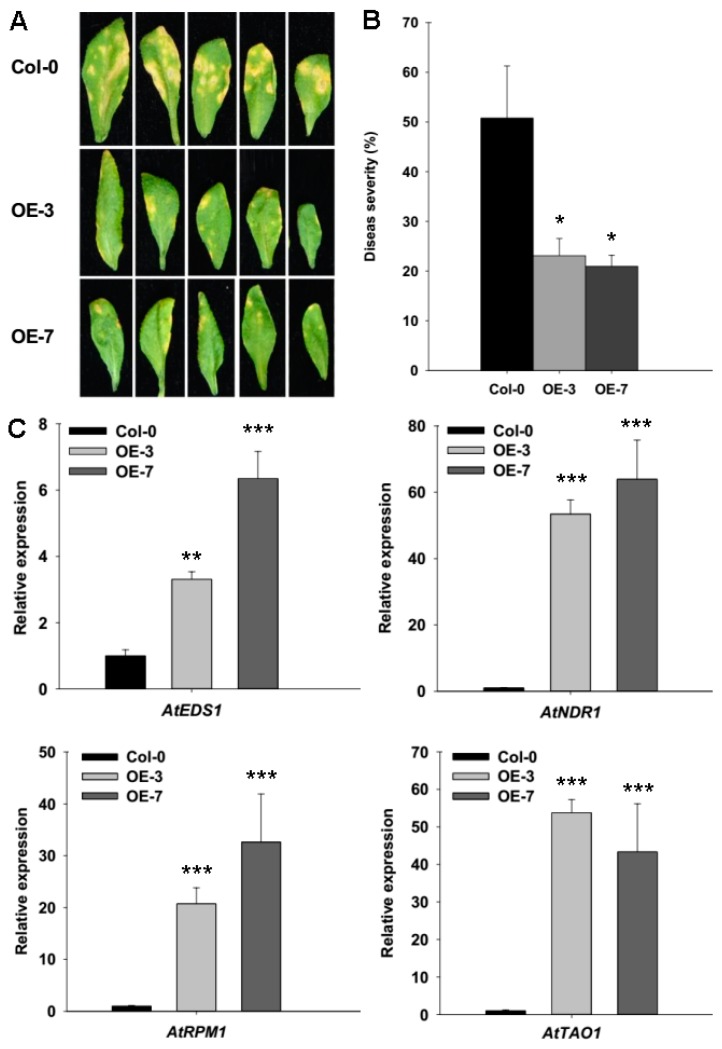
Enhanced resistance of *ZmRAD51A*-*OE* transgenic *Arabidopsis* plants to *Pst* DC3000. (**A**) Phenotypes of *ZmRAD51A*-*OE* and Col-0 leaves after *Pst* DC3000 infected for 7 days. (**B**) Disease index of *ZmRAD51A*-*OE* and Col-0 plants infected by *Pst* DC3000. (**C**) Expression levels of defense-related genes in *ZmRAD51A*-*OE* and Col-0 plants infected by *Pst* DC3000. Data were normalized using the transcript level of *AtUbiquitin* [36]. The expression levels of genes in Col-0 plants infected by *Pst* DC3000 were used as the control and assigned value of 1. OE-3 and OE-7 represents two different transgenic lines of *ZmRAD51A*-OE *Arabidopsis* plants. Student’s test was performed between Col-0 plants and *ZmRAD51A*-*OE* transgenic lines (* *p* < 0.05, ** *p* < 0.01 and *** *p* < 0.001). Data represent means SD. Three biological replicates were performed.

To investigate whether *ZmRAD51A* confers disease resistance to other species, we generated *ZmRAD51A* overexpression *Arabidopsis* plants. We firstly examined the impact of *ZmRAD51A* overexpression in *Arabidopsis* upon the infection of *Pst* DC3000 (*Pseudomonas syringae* pathovar *tomato* DC3000). The *ZmRAD51A*-OE lines showed significant resistance to *Pst* DC3000 at 7 dpi (Figure 6A,B). Col-0 plants had more lesions than *ZmRAD51A*-OE plants after *Pst* DC3000 infection (Figure 6A). The disease severity (DI, disease index) of *ZmRAD51A*-OE lines was 23.11% (OE-3) and 20.97% (OE-7) on average, but the Col-0 presented a DI of 50.78% at 7 dpi (Figure 6B). We further examined the expression levels of the genes that were involved in SA-dependent defense signaling pathway and pathogen resistance at 7 dpi, including *AtEDS1* (Enhanced Disease Susceptibility 1) [32], *AtNDR1* (Nonrace Specific Disease Resistance 1) [33], *AtRPM1* (Resistance to *P. syringae* 1) [34], and *AtTAO1* (Target of AvrB Operation) [35]. All the four genes that participated either in the SA signal pathway or in the resistance to *Pst* DC3000 pathogens were expressed significantly higher in the *ZmRAD51A*-OE plants than in the Col-0 plants (Figure 6C). 

## 3. Discussion

### 3.1. ZmRAD51A Is a Conserved DNA Repair Protein in Maize

The structure analyses on amino acid sequences and 3D model suggests that ZmRAD51A is conserved across plants to mammals. Notably, ZmRAD51A showed a close evolutionary relationship with OsRAD51A1, which can bind single and double stranded DNA, and promote homology dependent renaturation as well as strand exchange reactions [16], suggesting a similar role of ZmRAD51A in maize. Although the *ZmRAD51A* has been demonstrated to play important roles in meiotic recombination and DNA repair in maize, little is known about its function in disease resistance. In this study, for the first time, we isolated *ZmRAD51A* and functionally characterized its roles on disease resistance function in model plants. 

### 3.2. ZmRAD51A Is Involved in SA-Signal Defense Responses 

The expression patterns of *ZmRAD51A* showed *ZmRAD51A* is enriched in ears, roots, tassels, and filaments (Figure 2A), where mitosis and meiosis are more active, supporting that *ZmRAD51A* plays roles in the repair of DSBs. Although *ZmRAD51A* is usually less expressed in maize leaf, interestingly, it was significantly upregulated (up to 13-fold) within 1 h upon SA application (Figure 2B). Such a quick response was also observed in other resistance genes, including the bacterial blight disease resistance gene *Xa1* in rice [37] and the *Ralstonia solanacearum* resistance gene *AhRRS5* in peanuts [38]. SA is a well-known phytohormone signaling molecule involved in controlling the defense gene expression against disease [39]. The exogenous application of SA to a plant induces systemic acquired resistance (SAR), increases the expression of pathogeneses-related (*PR*) genes and enhances resistance to a broad range of pathogens [40,41,42]. Similar to those SA-dependent defense-related genes [31,43,44], our results suggested that *ZmRAD51A* may also involve in SA signaling pathways against pathogen infection. In addition, the transient overexpression of *ZmRAD51A* in *N. benthamiana* showed that it can induce a hypersensitive response, causing cell death and also the accumulation of H_2_O_2_ in hypersensitive responses (Figure 3), indicating that *ZmRAD51A* may be involved in reactive oxygen species (ROS) signaling against disease. These results together suggested that *ZmRAD51A* may be involved in SA-dependent cell death and disease resistance during pathogen infection.

### 3.3. ZmRAD51A Confers Disease Resistance in Transgenic Plants

Phenylalanine ammonia lyase (PAL) is a SA pathway-associated gene and a key enzyme that controls the biosynthesis of SA [45]. *OsPAL06* knockout mutant showed increased susceptibility to *M. oryzae* and developed typical leaf blast symptoms, accompanied by a reduction of the SA level [45]. In this study, *OsPAL06* increased significantly in *ZmRAD51A*-*OE* transgenic lines at 7 days after being inoculated with *M. oryzae* (Figure 5C), indicating *ZmRAD51A* conferred resistance to rice blast, accompanied by increasing of the SA level. Moreover, *ZmRAD51A* also enhanced *Arabidopsis* resistance to *Pst* DC3000, by triggering the expression of SA-dependent signal genes and defense-related genes, *AtEDS1* (Enhanced Disease Susceptibility 1), *AtNDR1* (Nonrace Specific Disease Resistance 1), *AtRPM1* (Resistance to *P. syringae* 1), and *AtTAO1* (Target of AvrB Operation) (Figure 6C). *AtEDS1* operates in the upstream of SA-mediated defenses and requires resistance to be mediated by several R genes in *Arabidopsis* [32]. The increased expression level of *AtEDS1* in *ZmRAD51A*-OE *Arabidopsis* indicated that *ZmRAD51A* is involved in SA-mediated defense pathway. It has been reported that *AtNDR1* was required for disease resistance signaling mediated by members of disease resistance proteins in *Arabidopsis* in response to infection by *P. syringae* [46], while *AtRPM1* and *AtTAO1* were conferring disease resistance in response to *Pst* DC3000 [34,35]. The up-regulation of *AtNDR1*, *AtRPM1*, and *AtTAO1* in *ZmRAD51A*-OE plants suggested that *ZmRAD51A* plays a positive role in resistance against *Pst* DC3000. Given that *ZmRAD51A* plays an important role in DNA recombination in maize [18], the dual roles of *ZmRAD51A* in disease resistant gene and DNA recombination suggest an interesting mechanistic link between defense response and DNA recombination, which is also supported by the previous reports of microbial pathogens causing DNA damage [47]. The abundance of double strand breaks is reduced by plant defense responses, suggesting that the mechanisms for activating DNA repair processes may share some similarity with the induction of *PR* genes [48,49]. More recently, DNA damage has also been found to be associated with SA signaling, where the increased expression of *PR* genes and the growth suppression of *Fusarium solani* were found in peas [50]. 

## 4. Materials and Methods

### 4.1. Plant Materials and Treatments

Maize B73 plants were grown in a greenhouse at 28 °C under a 16 h light/8 h dark photoperiod. Healthy maize seedlings at the three-leaf stage were sprayed with 1 × 10^–3^ M salicylic acid (SA). After 0h, 1 h, 3 h, 12 h, and 24 h, leaves were harvested and stored at –80 °C for subsequent RNA extraction. Other plants were further cultured and harvested roots, leaves, stems, tassels, filaments, husks, and ears were stored at –80 °C for subsequent RNA extraction.

### 4.2. Quantitative Real-Time Polymerase Chain Reaction (qRT-PCR) Analysis 

Total RNA from maize, rice, and *Arabidopsis* tissues was extracted by TRIzol (Thermo Fisher Scientific, Waltham, MA, USA). The reverse transcription kit (Roche Molecular Systems, Inc., Pleasanton, CA, USA) was used for cDNA synthesize. The SYBR green PCR master mix (Roche Molecular Systems, Inc., Pleasanton, CA, USA) was used for qRT-PCR reaction. The qRT-PCR was performed by Applied Biosystems 7300 (Applied Biosystems, Foster City, CA, USA) according to the manufacturer’s instruction. The qRT-PCR primers used in this study are listed in Table S1. The relative expression levels of each gene were calculated by formula 2^−ΔΔCt^ [38].

### 4.3. Bioinformatics Analysis

The *ZmRAD51A* gene structure was analyzed by GSDS (Gene Structure Display Server; http://gsds.cbi.pku.edu.cn/) [51]. The 3D structure of ZmRAD51A was assembled by SWISS-MODEL (https://swissmodel.expasy.org/) according to homology modeling, using RAD51 from humans as a template [52]. The PDB IDs of RAD51 is 5jzc.1. A. Proteins sequences between ZmRAD51A and other RAD51 were aligned by MEGA6 [53]. A phylogenetic tree was then constructed by the neighbor-joining method (bootstrap = 1000) [54]. Gene transcription data of *ZmRAD51A* in various growth time and tissues was used to draw a heat map by R/Bioconductor (http://www.bioconductor.org/) [55]. Promoters were analyzed by RSAT (http://floresta.eead.csic.es/rsat/) [56]. 

### 4.4. Full-Length cDNA Cloning and Vector Construction

The full-length cDNA of *ZmRAD51A* was isolated from maize leaves cDNA by using PCR with specific primers (Forward ATGGCAGAAGCTGTGGTGTT and reverse CTATATGCGCAACTCCAGACC) and PrimeSTAR Max DNA Polymerase (TaKaRa Bio Inc., Kusatsu, Shiga, Japan). Then, the products were cloned and sequenced. The full-length cDNA of *ZmRAD51A* was constructed into the pCAMBI1301vector to obtain a p35S:*ZmRAD51A* fusion gene.

### 4.5. Cell Death Assays in Nicotiana Benthamiana

*Agrobacterium tumefaciens* strain GV3101 containing 35S:*ZmRAD51A* and pCAMBI1301 (control) constructs were respectively injected into *N. benthamiana* leaves by syringe, which contained the volume of about 100 μL as described by Stella et al. [57]. Photos of *N. benthamiana* leaves phenotype were taken 48 h after Agrobacterium infection. DAB and trypan blue staining were performed as previously described [38,58]. *N. benthamiana* leaves were treated with DAB (3′-Diaminobenzidine, Sigma, St. Louis, MO, USA) solution (1 mg mL^−1^) overnight and then cleared with 95% ethanol. For trypan blue staining, the leaves were boiled in lactophenol-trypan blue solution (1 mg mL^−1^) for 5 min and destained overnight in chloral hydrate (2.5 g mL^−1^). The DAB and trypan blue staining leaves were observed under a microscope (Leica DM5000 B, Leica Microsystems Ltd., Heerbrugg, Switzerland). 

### 4.6. Rice Transformation

The seeds of rice Zhonghua 11 were used to induce embryogenic calli. Well growth embryogenic calli were harvested for infection by *A. tumefaciens* GV3101 harboring the 35S:*ZmRAD51A* constructs and pCAMBI1301 (control) as previously described [37]. After 2 days co-cultivated with *A. tumefaciens* GV3101 in N6 medium containing 200 μM acetosyringone, embryogenic calli were thoroughly washed with ddH_2_O, and then transferred to a new N6 medium, containing 0.25 g mL^−1^ cefotaxime and 0.05 g mL^−1^ hygromycin. After several selections, the embryogenic calli propagated plants in regeneration medium. 

### 4.7. Measurement of SA

SA extraction and quantification were performed according to the method described in the previous study [59,60]. The leaves of rice transformed of pCAMBI1301 and *ZmRAD51A* were excised and frozen in liquid nitrogen (N_2_). The SA was extracted from 0.3 g leaf powder by sequentially subjecting them to 70% and 90% methanol. The 2-methoxybenzoic acid (Sigma-Aldrich, St. Louis, MO, USA) was used as an internal SA standard. The reverse-phase high-performance liquid chromatography (RP-HPLC) (Agilent 1200 series with a C18 column [150 mm × 4.6 mm, 5 μm], Agilent Technologies, Santa Clara, CA, USA) was used for SA measurement. 

### 4.8. Rice Pathogens Inoculation

*ZmRAD51A* overexpression rice and control of rice plants with the fourth leaf fully expanded were selected for disease analysis. Rice seedlings were inoculated with *Magnaporthe oryzae* Guy 11 using the spraying method to assess the resistance of transgenic rice to blast [61]. Disease was scored by measuring the percent lesion area (lesion length/leaf length) at 7 days after inoculation [31].

### 4.9. Arabidopsis Transformation

*Arabidopsis* plants Col-0 were transformed by *A. tumefaciens* GV3101 carrying the 35S:*ZmRAD51A* using the floral dip method [62]. Transformed *Arabidopsis* seeds were screened on Murashige and Skoog plates containing 20 mg mL^−^^1^ hygromycin. DNA was extracted from the *Arabidopsis* plant using the CTAB (cetyl trimethyl ammonium bromide) method [63], which was used as a template for PCR to further determine positive transgene integration. The 2× Taq Master Mix (Dye Plus; Vazyme Biotech Co. Ltd., Nanjing, China) was used for the PCR reaction. After several screens, the homozygous T3 generation was used for experiments.

### 4.10. Arabidopsis Pathogen Inoculation

*Pseudomonas syringae* pv. (pathovar) *tomato* DC3000 (*Pst* DC3000) was cultured at 28 °C on King’s B (KB) medium. After 2 days, a single *Pst* DC3000 colony was inoculated in 5 mL KB medium for 1 day at 28 °C. One milliliter of the culture was inoculated in 100 mL KB medium for 1 day. The bacterial was harvested and suspended in a solution which included 0.01% Silwet L-77 and 10 mM MgSO_4_ with final OD_600_ = 1.0. Two transgenic *Arabidopsis* lines (OE-3 and OE-7) and Col-0 were sprayed with *Pst* DC3000 suspension. Whole *Arabidopsis* plants were sprayed by the bacterial suspension and then covered with plastic film for 3 days. Disease index was evaluated after 7 days post inoculation (dpi) as Niu et al. described [64]. Three biological replicates were set.

### 4.11. Statistical Analyses

The data were subjected to excel software 2016 with analysis of student’s test to evaluate differences between control and treatment samples. Statistical significance was set at * *p* < 0.05, ** *p* < 0.01 and *** *p* < 0.001.

## Supplementary Materials

Supplementary materials can be found at http://www.mdpi.com/1422-0067/20/4/807/s1.

## Abbreviations

qRT-PCR	Quantitative real-time polymerase chain reaction
SA	Salicylic acid
HPLC	High-performance liquid chromatography
PTI	PAMP-triggered immunity
ETI	Effector-triggered immunity
NPR1	NONEXPRESSOR OF PR1 GENES
DSBs	DNA double strand breaks
SAR	systemic acquired resistance
