# Supplementary material for: DNA Repair Gene ZmRAD51A Improves Rice and Arabidopsis Resistance to Disease
Source: Int J Mol Sci. 2019 Feb 13;20(4):807. doi: 10.3390/ijms20040807 (PMC6412738; doi:10.3390/ijms20040807)
Supplement: Supplementary file 1 [file ijms-20-00807-s001.pdf]

DNA Repair Gene *ZmRAD51A* Improves Rice and Arabidopsis Resistance to Disease

Supplementary materials

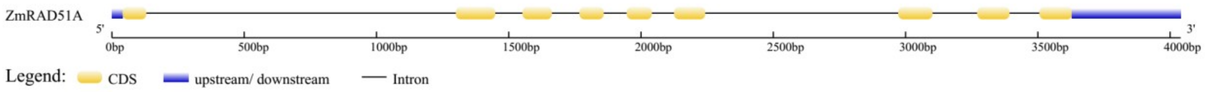

Figure S1. Gene structure of *ZmRAD51A.*

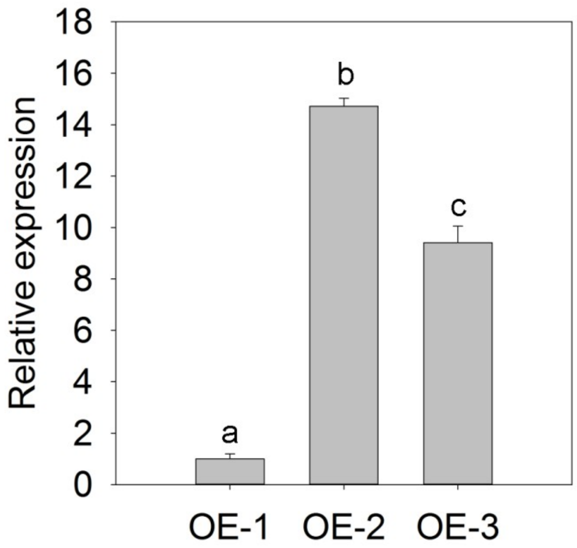

Figure S2. Relative expression levels of *ZmRAD51A* in transgenic rice lines. Different letters above the columns represent significant differences among three lines at $P < 0.05$.

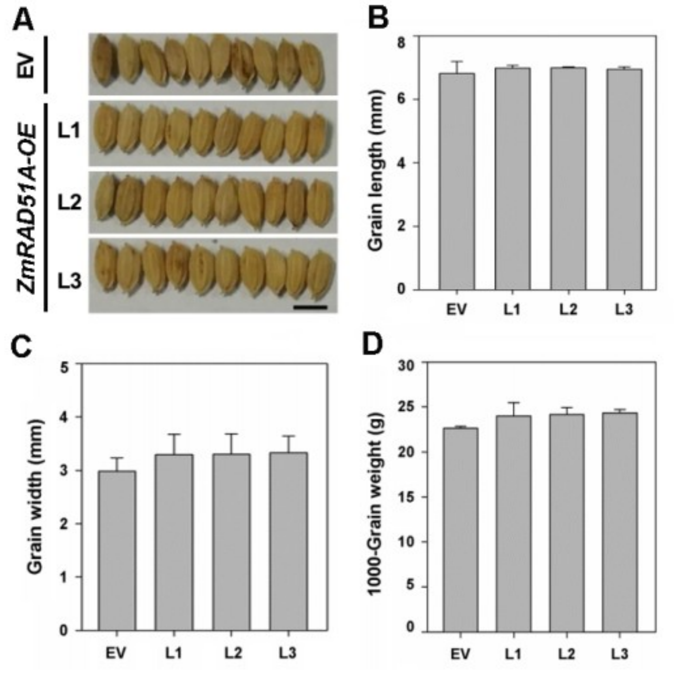

Figure S3. Grain phenotypes of *ZmRAD51A-OE* transgenic rice lines. (A) Comparison of grain size between the *ZmRAD51A-OE* and pCAMBIA1301 (EV) transgenic rice lines. (B) Grain lengths of the *ZmRAD51A-OE* and pCAMBIA1301 (EV) transgenic rice lines seeds. (C) Grain width of the *ZmRAD51A-OE* and pCAMBIA1301 (EV) transgenic rice lines seeds. (D) 1000-grain weights of the *ZmRAD51A-OE* and pCAMBIA1301 (EV) transgenic rice lines seeds. Scale bars (A) = 5 mm. Data represent means SD. Three biological replicates were performed.

Table 1. Primers used for qRT-PCR in this study.

Primer names	Primer sequences
RT-AtEDS1-F	AAGACCAACCCGCTACATA
RT-AtEDS1-R	TTCAACCTCAGCCCAAAAG
RT-AtNDR1-F	GAAGTCAACGGTGATGGAGT
RT-AtNDR1-R	AACGAATAGCAAAGAATACGA
RT-AtTAO1-F	GACCGTTTTTATGATGTGG
RT-AtTAO1-R	CGAAGTTATCTCCGTCGTAT
RT-AtRPM1-F	GGTTGTTCCTAAAATCTGGC
RT-AtRPM1-R	TCTAACGGTTCCTCTTCATC
RT-AtUbiquitin-F	CTGCGACTCAGGGAATCTTCTAA
RT-AtUbiquitin-R	TTGTGCCATTGAATTGAACCC
RT-OsPAL06-F	CGTCCGCATCAACACACTCC
RT-OsPAL06-R	CGTTCACCTTGCTGCCATCT
RT-OsPIKM-F	TGATGATAAGACATCGGTCAGG
RT-OsPIKM-R	TCATTTTCAGAAGGCAGAAACG
RT-OsActin-F	TCCATCTTGGCATCTCTCAG
RT-OsActin-R	GTACCCGCATCAGGCATCTG
RT-ZmRAD51A-F	GTGCCAGTCAACTTCATGCG
RT-ZmRAD51A-R	TTTCCAGAGCGGAACTCACC
RT-ZmActin-F	GGGATTGCCGATCGTATGAG
RT-ZmActin-R	GAGCCACCGATCCAGACACT
